# Mortality in people experiencing homelessness: a systematic review and meta-analysis

**DOI:** 10.1093/ije/dyag129

**Published:** 2026-09-18

**Authors:** James White, Yvonne Moriarty, Mandy Lau, Rebecca Cannings-John, Abigail Palmer, Alison L Weightman, Meg Kiseleva, G David Batty

**Affiliations:** Centre for Trials Research, School of Medicine, Cardiff University, Cardiff, United Kingdom; Centre for the Development, Evaluation, Complexity and Implementation in Public Health ImpRovement (DECIPHer), School of Social Sciences, Cardiff University, Cardiff, United Kingdom; Centre for Trials Research, School of Medicine, Cardiff University, Cardiff, United Kingdom; Centre for Trials Research, School of Medicine, Cardiff University, Cardiff, United Kingdom; Centre for Trials Research, School of Medicine, Cardiff University, Cardiff, United Kingdom; Centre for the Development, Evaluation, Complexity and Implementation in Public Health ImpRovement (DECIPHer), School of Social Sciences, Cardiff University, Cardiff, United Kingdom; Specialist Unit for Review Evidence (SURE), Cardiff University, Cardiff, United Kingdom; Specialist Unit for Review Evidence (SURE), Cardiff University, Cardiff, United Kingdom; Department of Epidemiology and Public Health, University College London, London, United Kingdom

**Keywords:** homelessness, mortality, cause-specific mortality, rough sleeping, shelter

## Abstract

**Background:**

The association between homelessness and the type of homeless experience with all-cause and cause-specific mortality is uncertain.

**Methods:**

Published studies were retrieved through a systematic search of MEDLINE, Embase PsycINFO, and Scopus from inception to December 2024. Unpublished data were identified from open-access data archives. We used random-effects meta-analysis to combine the effect estimates from published and unpublished data from 116 studies comprising 2 563 633 homeless people and 129 292 553 population controls. This review is registered at PROSPERO (CRD42023430984).

**Results:**

The risk of all-cause mortality was increased in people exposed to homelessness compared with those unexposed [relative risk (RR) 2.21, 95% confidence interval (CI) 1.91–2.57, *P* < .001, *I*^2^ = 99.7], with risks similar in men (RR 4.04, 95% CI 2.87–5.21) and women (RR 3.58, 95% CI 2.35–4.82). Risks were higher in people who had slept rough (RR 7.63, 95% CI 3.29–11.97) than in those who had slept in homeless hostels/shelters (RR 3.44, 95% CI 2.10–4.77) and also elevated for people staying in low-cost hotels (RR 5.18, 95% CI 1.14–9.23). Summary RR estimates were elevated for 33 of the 36 (92%) causes of death. The highest risk of death was apparent for psychoactive-substance-use disorder (RR 21.38, 95% CI 14.44–31.67), accidental injuries (RR 13.15, 95% CI 5.46–31.69), and drug overdose (RR 11.65, 95% CI 6.97–19.48). Around half of the studies (57%) were of high quality. No studies were found in low-income countries. There was no evidence of publication bias.

**Conclusion:**

Compared with the general population, homeless people experience an increased risk of premature mortality. The most extreme inequalities were from deaths through psychoactive-substance-use disorder, accidents, and drug overdose. There are evidence-based treatments for psychoactive substance use and drug overdose, suggesting that some of these deaths are avoidable.

Key Messages

- We sought to determine whether homelessness was associated with the risk of all-cause and cause-specific mortality.
- We found that people exposed to all forms of homelessness had an elevated risk of all-cause mortality and were more likely to die from psychoactive-substance-use disorder, drug overdose, accidental injury, and mental illness compared with people who were not exposed to homeless.
- Homelessness and many of these causes of death are eminently avoidable.

## Introduction

Worldwide, it is estimated that nearly 1.6 billion people have inadequate shelter, with ∼15 million people forcefully evicted every year [1]. In low- and middle-income countries, a combination of increasing urbanization and forced displacement from conflict and climate change is increasing the number of people without permanent shelter [2–4]. In high-income countries, insufficient social housing and increasingly unaffordable private housing are leading to a surge in homelessness. An estimated 895 000 people in Europe—one in every 600 people—sleep rough in shelters or temporary accommodation every night, which is an increase of 40% since 2023–4 [5]. Corresponding data from the USA suggests that 771 480 people are currently experiencing homelessness, which is an increase of 18% on 2023–4, with the majority living in emergency shelters or temporary accommodation such as single-occupancy low-cost hotels [6].

While existing studies have provided an important evidence base [7, 8], gaps hamper their usefulness for policy makers, as the types of homeless experience, population subgroups, and causes of death that are the most elevated are unclear. First, in the only previous meta-analysis, the authors combined the results from three studies with people who had been homeless with people who had been incarcerated, were sex workers, or had a substance-use disorder, such that no estimate of the association between homelessness and all-cause mortality was reported [7]. Second, little is known about whether the risk varies according to the characteristics of the individuals (e.g. sex at birth), type of homeless experience (e.g. rough sleeping, shelters, low-cost hotels), or country of residence. Third, individual studies have summarized associations for a small number of causes of death [9, 10], presented cause-specific estimates for a single country (which may limit generalizability) [11, 12], or focussed on high-income regions [9–12] or one type of homeless experience [12]. Lastly, publication bias—the notion that studies reporting positive results are more likely to be published—has not been explored and can distort the evidence base.

This study aimed to address these gaps by conducting a comprehensive review of the global evidence on homelessness with all-cause and cause-specific mortality. The primary estimates that we chose are those most adjusted and those using socio-economically deprived comparator groups, as these are likely to be the best estimates of the effect of homelessness on mortality.

## Methods

The review was prospectively registered with PROSPERO (CRD42023430984) [13] and conducted in accordance with a published protocol [14]. We followed the PRISMA-IPD [15] and MOOSE [16] reporting guidelines in the presentation of our report.

### Search strategy and selection criteria

We identified published studies through a systematic search of MEDLINE, Embase, PsycINFO, and Scopus from their inception to December 2024. Full search terms are provided in the Supplementary file (Supplementary Table S1). No limits on language were applied and articles were translated into English if required.

Full texts were then retrieved of papers that appeared to meet the inclusion criteria or, where a decision could not be made, based on titles and abstracts. We also conducted a supplementary search of the reference sections of systematic reviews and websites to identify grey literature.

After the exclusion of duplicate studies, M.K. and A.W. independently reviewed all titles and abstracts to establish their eligibility on the basis of defined inclusion criteria [14]. As definitions of homelessness vary internationally [17, 18], we used an inclusive definition of people who are, or report ever having been, homeless. This included people with different types of homeless experience: (i) sleeping rough (e.g. living directly on the streets, in squats); (ii) staying in an emergency hostel, shelter, or refuge for homeless people; (iii) residing in low-cost hotels (e.g. temporary accommodation including single-occupancy rooms, bedsits, boarding houses); or (iv) ‘sofa (couch)-surfing’ (e.g. staying with friend(s) or relative(s); herein called sofa-surfing). We excluded studies in which a residence was (or had been) threatened due to financial insecurity; if the people resided in poor living standards; or if the authors provided insufficient information to calculate an estimate, such as those studies solely quantifying mortality in a homeless population without a comparator group.

Any disagreements were resolved through discussion, involving a third reviewer where necessary. Where we identified duplicate data presented in separate publications, we selected the study with the largest or most representative sample size and, when these were also similar, we used the most recently published report.

### Data extraction

All authors extracted the data, which was then double-checked. Extracted characteristics included: name of the first author, publication year, study location (country), study type, homelessness type, current or historical homelessness, comparator population, degree of socio-economic disadvantage (e.g. occupational social class, income), follow-up period, number of people at risk and number of deaths in exposed and non-exposed groups, the summary statistics reported for age and sex (e.g. either reported as frequency, percentage, or mean or median alongside standard deviation or interquartile range), and the extent of statistical adjustment.

### Unpublished individual-participant data

We supplemented data from the published studies with unpublished individual-level data from prospective cohort studies, located by searching two websites on which we had previously found data on homelessness and mortality [32], the UK Data Service (https://ukdataservice.ac.uk), and the Inter-University Consortium for Political and Social Research (http://www.icpsr.umich.edu/icpsrweb/ICPSR/). This yielded two studies: the Survey of Health, Ageing and Retirement in Europe [19] and the US Health & Retirement Study [20]. Both had approvals from ethics committees and all participants had given informed consent. The harmonized covariates in our analyses of unpublished studies included age, sex, and socio-economic status.

### Outcomes

Outcomes were all-cause and cause-specific mortality defined according to the International Classification of Diseases, tenth revision (ICD-10).

### Quality assessment

To assess the quality of the included studies, we used the MethodologicAl STandards for Epidemiological Research (MASTER) scale [21]. Assessments were made of: formal recruitment, equal retention, equal ascertainment, equal implementation, equal prognosis, sufficient analysis, and temporal precedence [minimum score = 0, maximum (high quality) = 36]. The quality of the study was regarded as having a low risk of bias if the total score was above a median of 12.

### Statistical analysis

The statistical analysis plan was completed before analysis (Supplementary file, page 6). The data and code for all the statistical analyses can be found at GitHub (https://github.com/jameswhite1979/Homelessness-systematic-review). The data describe the causes of death investigated by each study. We first analysed unpublished individual-participant data by using Cox proportional-hazards regression to generate hazard ratios (HRs) with accompanying 95% confidence intervals (CIs) for the associations between homelessness and all-cause and cause-specific mortality [22].

Next, we used meta-analysis to combine the results from the analyses of the unpublished data with estimates from published studies. Where published studies provided HRs and odds ratios (ORs), these were used as a common estimate of relative risk (RR). In studies in which the incidence of mortality was <10%, we regarded the ORs as a close approximations of RR [23]. If the mortality incidence was ≥10% and count data were available, then ORs and HRs were converted into RRs [24, 25]. If CIs were missing, then they were calculated by using standard formulae.

We analysed the associations of homelessness with all-cause and cause-specific mortality separately. For the unpublished data, effect estimates were adjusted for age and sex, and socio-economic status. For published studies, we used the most comprehensively adjusted estimates. Some publications reported multiple effect sizes, creating a unit-of-analysis issue. To account for this, when multiple comparator population groups were reported, we used estimates where the comparison group was a socially deprived population or where measures were adjusted for area, household, or individual-level deprivation. If studies reported multiple comparisons (e.g. short and longer follow-ups), we chose the comparison with the greatest number of deaths. Where multiple exposures were compared with the same comparator group (e.g. different types of homelessness to the same unexposed population), they were combined. Where the exposed group was compared with more than one comparator group (e.g. other cohort participants or an external population), we ran a multilevel meta-regression to model the effects nested within studies to account for the association between the common groups [26].

We used random-effects models reflecting the anticipated heterogeneity in the included studies. We used the *I*^2^ statistic to summarize the proportion of total variation in study estimates due to heterogeneity, with higher values denoting greater heterogeneity [27]. Planned exploratory subgroup analyses were conducted according to participant sex, country, homelessness status (current vs. historical), type of homeless (sleeping rough vs. shelter vs. temporary accommodation), and according to the income of the country from which the study sample was drawn based on the World Bank 2023 classification [28]. We also examined differences according to the study characteristics, including exposure ascertainment (self-report vs. administrative record), comparator population (socio-economically deprived vs. general; internal vs. external cohort), effect sizes adjustment (minimal vs. multivariable), publication status (unpublished vs. published), study design (retrospective vs. prospective), total sample size (<1000, 1000 to <10 000, 10 000 to <100 000, 100 000 to <500 000, 500 000 to <1 million, 1 million to <5 million, 5 million to <10 million, ≥10 million), and study quality (categorized as low, high). Subgroup differences were estimated by using meta-regression.

Summary RRs for causes of death were first estimated at the ICD chapter level (e.g. cancer, external) and then at higher specific-cause resolution by using the descriptors provided by the authors (e.g. lung cancer, suicide). Publication bias was explored by using an analysis of asymmetry in funnel plots [29]. Where detected, we used the non-parametric trim-and-fill method to quantify its effect [30]. As we anticipated retrieving a small number of very large studies, we used the ‘leave-one-out’ method [31] to examine the impact of excluding each on the summary effect size.

We performed statistical analyses by using Stata (MP version 18) to analyse the unpublished data and Stata (MP version 18) and R (version 4.3.3) to compute the meta-analysis.

## Results

**Figure 1:**
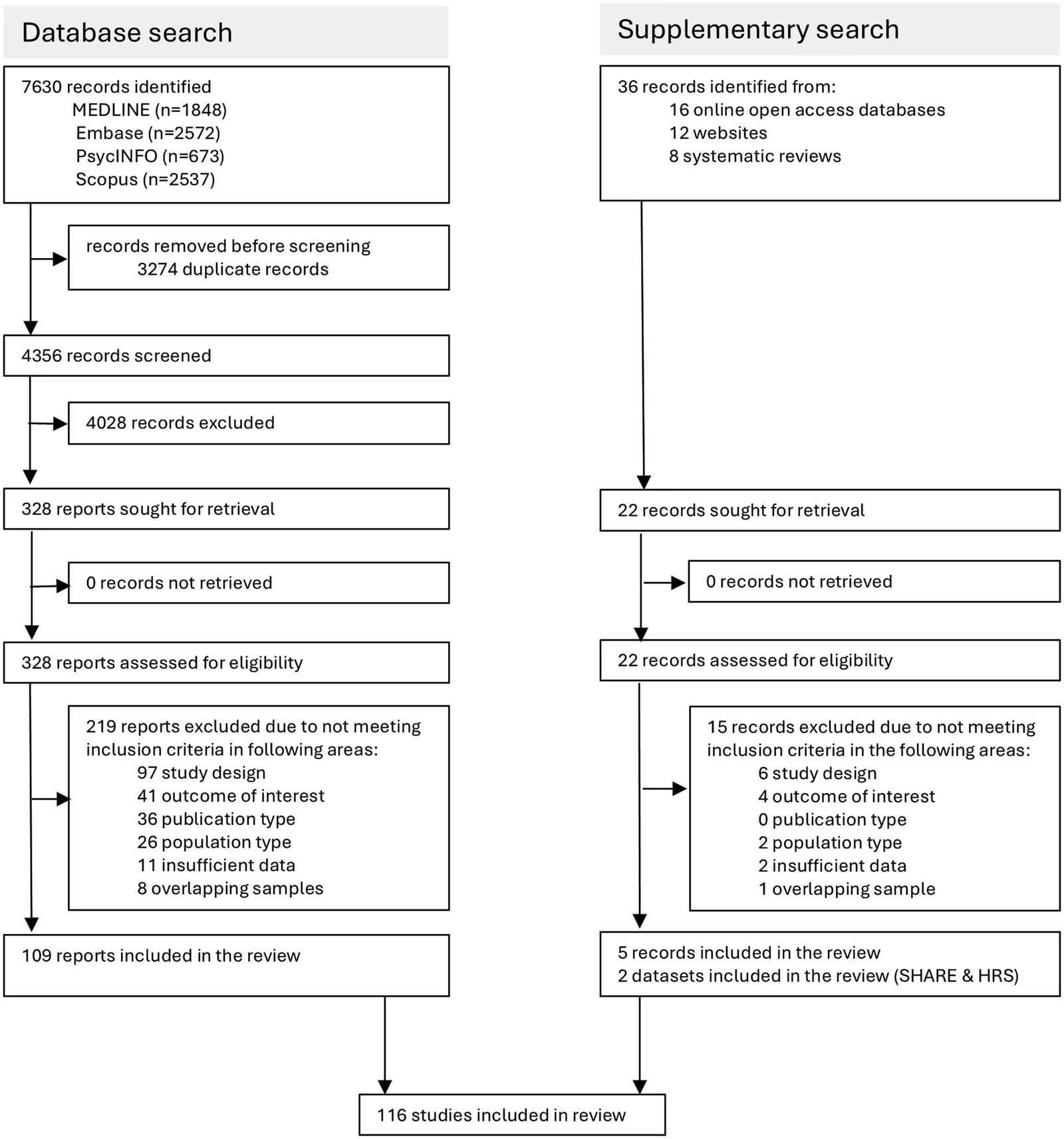
Study selection.

Figure 1 depicts the process of study selection. We identified 7630 records from four electronic database searches, of which 3274 were duplicated. In a parallel supplementary search for eligible unpublished datasets and grey literature, we scrutinized the reference sections of 8 systematic reviews, 12 websites, and 16 online databases. In so doing, 36 additional records were identified. We reviewed the full texts of 350 studies, of which 116 (114 published plus 2 unpublished datasets, as described in Supplementary Table S2) met our inclusion criteria, comprising ≥131.9 million people. Excluded studies and reasons for exclusion with study counts are listed in Supplementary Table S3.

A total of 1 618 049 people who were homeless and an estimated 109 274 222 from comparator populations contributed to the meta-analysis of all-cause mortality. Ninety-five studies yielded 126 unique RRs in the analyses of all-cause mortality, of which the large majority (91.3%, *N* = 115) had an elevated risk of death in the homeless group. Homelessness was associated with a doubling in the risk of all-cause mortality (RR 2.21, 95% CI 1.91–2.57, *P* < .001; *I*^2^ = 99.7%; *P* < .001, Supplementary Figure S1). Just over half of the studies (57.1%) were evaluated as being of high quality. We found no strong evidence of publication bias (Egger’s test value = 1.86, *P* = .06; Supplementary Figure S2).

**Figure 2:**
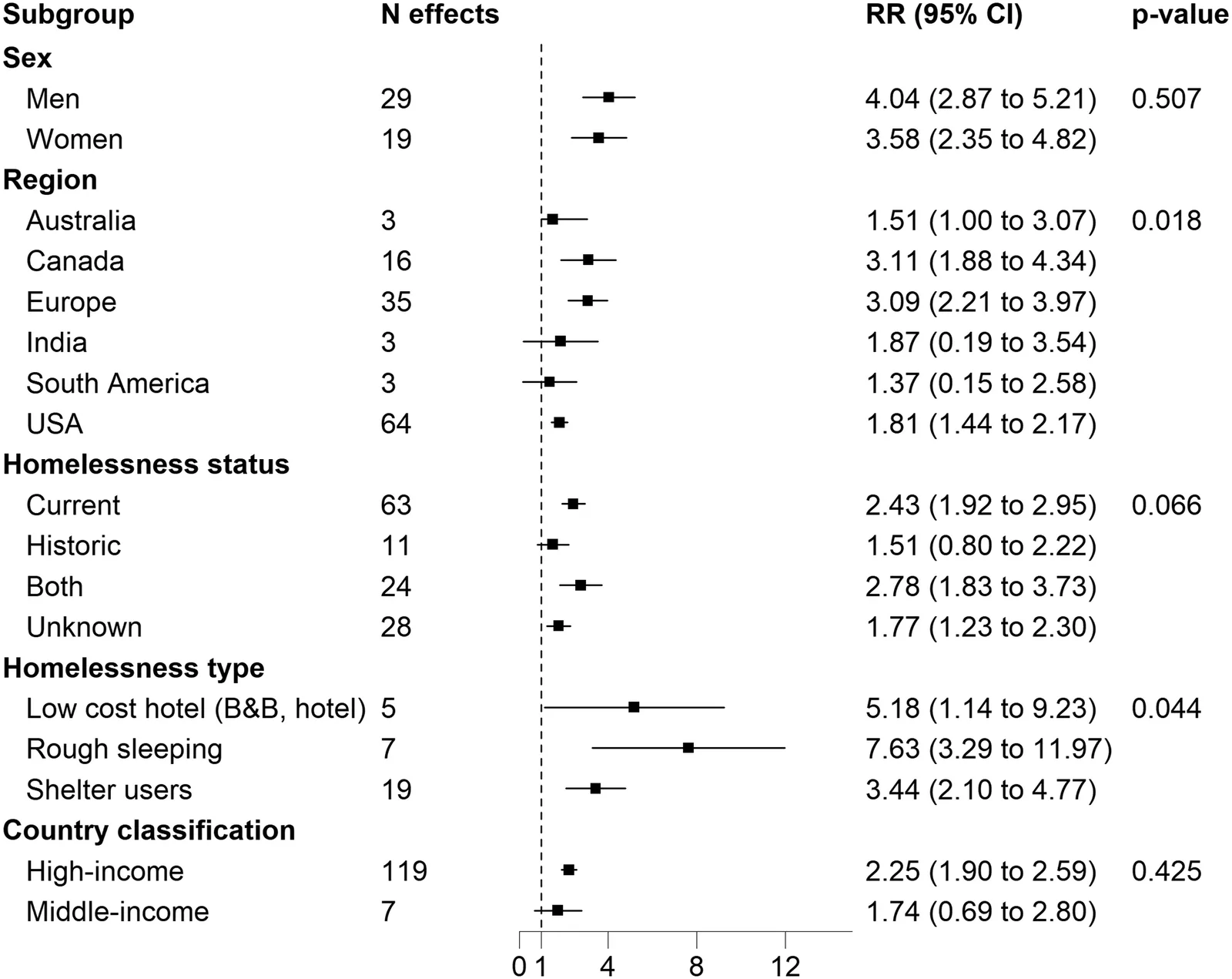
Association between homelessness and all-cause mortality comparing population subgroups.

There were no marked differences in the homelessness–mortality association between men (RR 4.04, 95% CI 2.87–5.21) and women (RR 3.58, 95% CI 2.35–4.82) or whether their experience was current (RR 2.43, 95% CI 1.92–2.95) compared with historical (RR 1.51, 95% CI 0.80–2.22). A stronger association was apparent in people who had slept rough (RR 7.63, 95% CI 3.29–11.97) compared with in those using a shelter (3.44, 95% CI 2.10–4.77, *P* for difference = .01) and residence in a low-cost hotel was also associated with an elevated mortality risk (RR 5.18, 95% CI 1.14–9.23) but, as only one study was found to have reported on sofa-surfing [32], we did not report on it here. There were also differences in effect estimates by geographical location, with higher RRs in samples drawn from Europe (including the UK) relative to the USA (*P* for difference = .02) and Canada compared with the USA (*P* = .003, Fig. 2).

**Figure 3:**
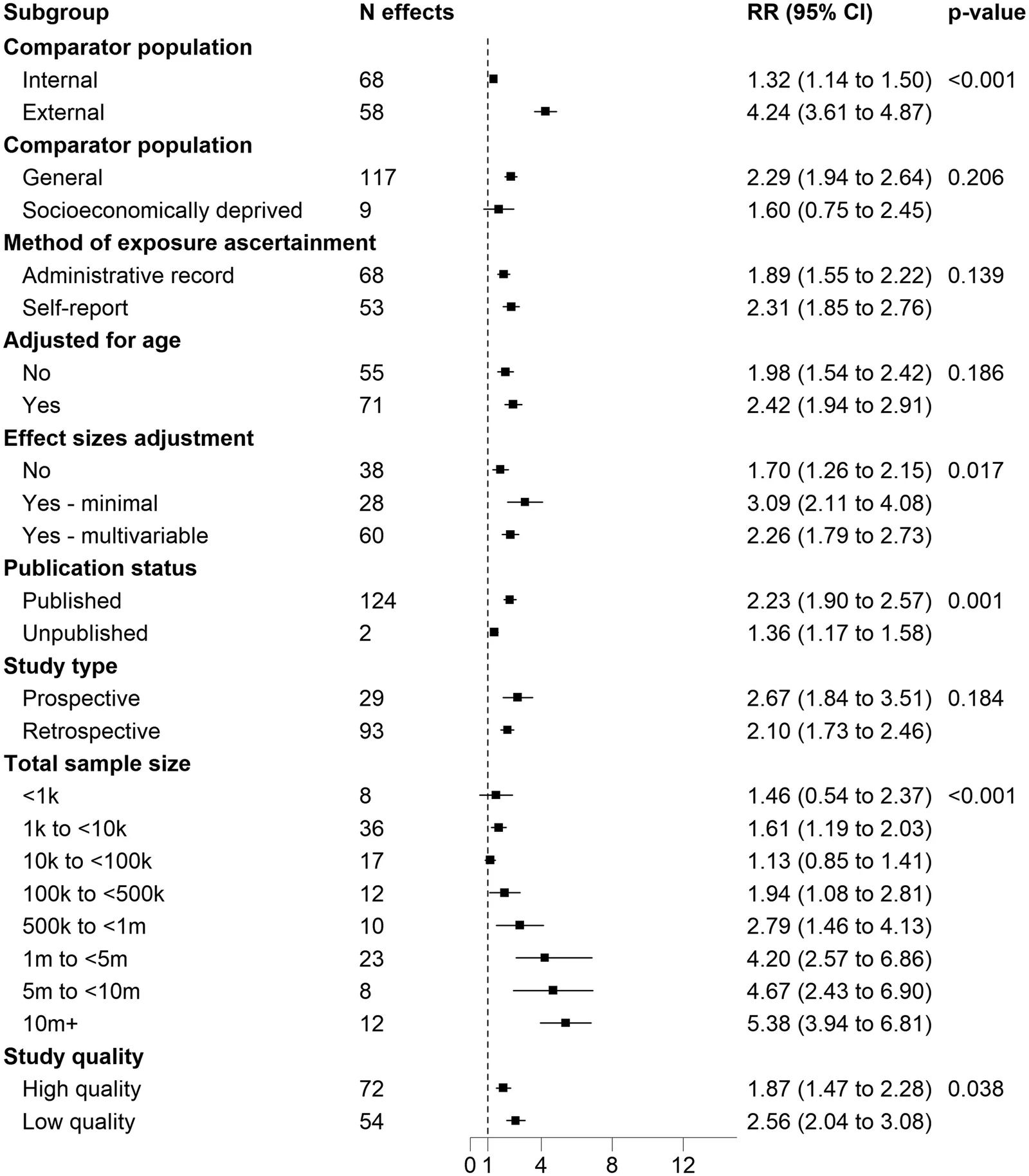
Association between homelessness and all-cause mortality comparing study characteristics.

We examined the methodological characteristics of the studies finding no strong evidence of bias arising from the type of comparator population used; the method of exposure ascertainment; whether age was adjusted for in the model or not; or the study design. Summary RRs were higher in studies that used an external compared with an internal comparator population; in studies that provided adjusted compared with unadjusted estimates; in lower-quality studies; and in studies with larger sample sizes (*P* for trend for increasing sample size < .001); published studies had larger effects than unpublished studies (Fig. 3).

**Figure 4:**
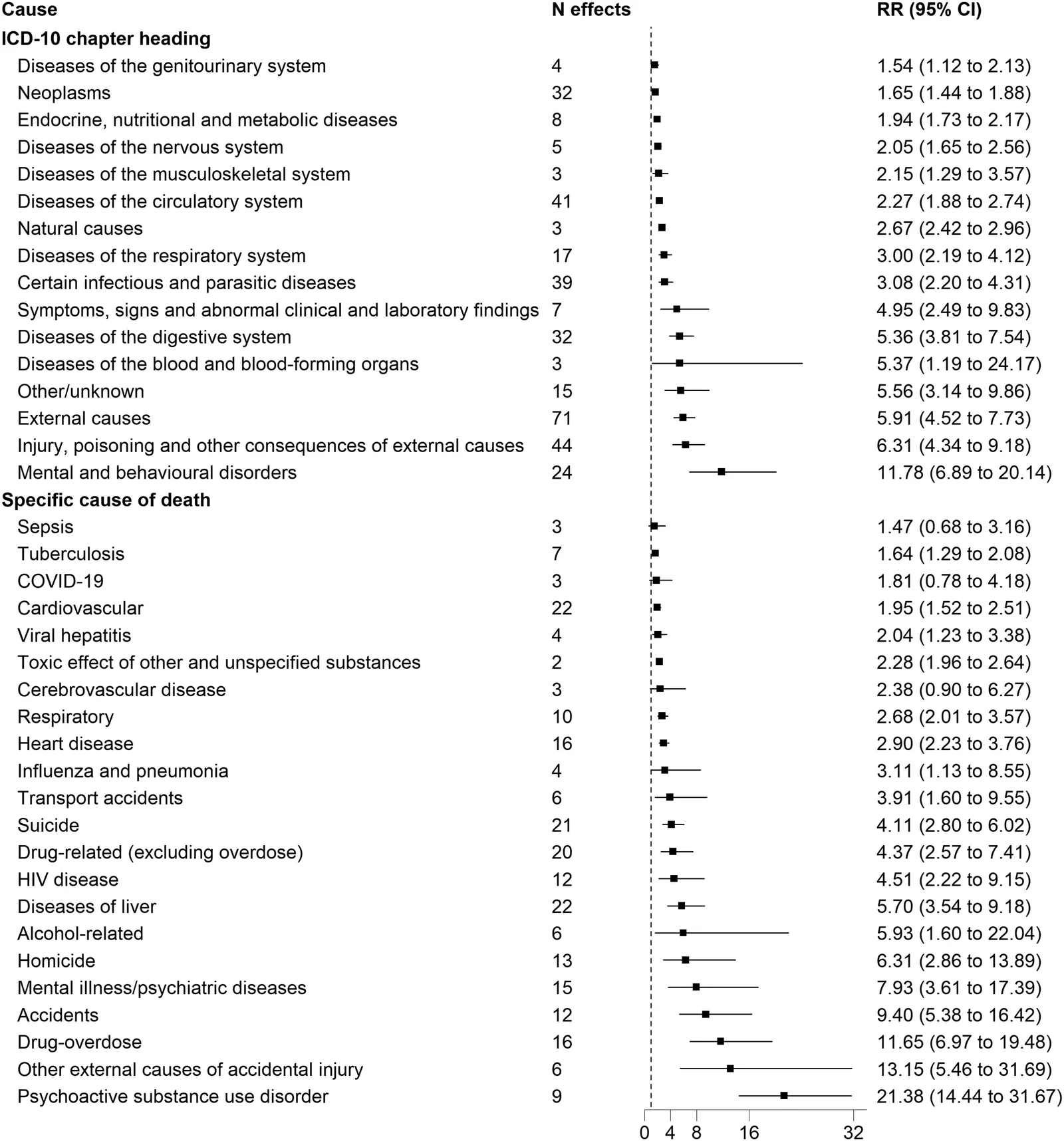
Association between homelessness and cause-specific mortality according to the ICD-10 disease categories.

Summary estimates for cause-specific mortality were elevated for 33 of the 36 (92%) causes of death investigated (*n* homeless = 1 202 205; *n* comparator = 25 089 695, Fig. 4). At the ICD-10 chapter level, summary RRs were particularly high for mental and behavioural disorders (11.78, 95% CI 6.89–20.14, F00–F99 in ICD-10); injury, poisoning, and other consequences of external causes (6.31, 95% CI 4.34–9.18, S00–T98 in ICD-10); other/unknown deaths (5.56, 95% CI 3.14–9.86, R00–R99 in ICD-10); diseases of the blood and blood-forming organs (5.37, 95% CI 1.19–24.17, D50–D89 in ICD-10); and external causes (5.91, 95% CI 4.52–7.73, V01–Y98 in ICD-10).

Summary RRs for disaggregated individual causes were the highest for deaths attributed to psychoactive-substance-use disorder (21.38, 95% CI, 14.44–31.67); drug overdose (11.65, 95% CI 6.97–19.48); other external causes of accidental injury (13.15, 95% CI 5.46–31.69); accidents (9.40, 95% CI 5.38–16.42); alcohol-related causes (5.93, 95% CI 1.60–22.04); drug-related (excluding overdose) (4.37, 95% CI 2.57–7.41); homicide (6.31, 95% CI 2.86–13.89); and suicide (4.11, 95% CI 2.80–6.02) (Fig. 4).

Sensitivity analyses using the leaving-one-study-out approach to explore the effect of individual studies had little impact on the majority of findings. Excluding one study conducted in the UK [33], with Standardised Mortality Ratios (SMRs) 2–3.7 times larger than those of any other study, reduced the pooled RR for Europe from 3.09 (95% CI 2.21–3.97) to 2.39 (95% CI 1.84, 3.11). The authors of this study acknowledged uncertainty in the denominator for the homeless group such that the SMRs may have been inflated.

## Discussion

Our systematic review and meta-analysis found a doubling in the risk of death in people with a history of homelessness relative to a general comparator population. The magnitude was comparable for men and women, by current or historical exposure, and elevated across all forms of homelessness, including rough sleeping and residence in a low-cost hotel or shelter, and higher in studies drawn from Canada compared with the USA. We found that the mortality was elevated for almost all health conditions examined, with some extreme RRs of >20 for psychoactive-substance-use disorder, 10–19 for drug overdose and external causes of accidental injury, and 5–10 for accidents and alcohol-related causes.

Our findings are consistent with those of the previous systematic reviews of homelessness and all-cause mortality [7, 8] that included a substantially smaller evidence base (11 studies) as well as a comparatively large study with 6.3 million people conducted in a single high-income county [12]. In the present review, we included 116 studies comprising at least 131.9 million people. Consistently with the one previous study on the type of homelessness [32], we found that mortality was increased across all types, including low-cost hotels. This is likely to be of interest to policy makers, as low-cost hotels have recently been used in high-income countries to house single adults [34] and asylum seekers [35, 36] over long periods when settled accommodation is not available. Importantly, we provided a comprehensive assessment of the association between homelessness across 36 causes of death [7, 8]. The evidence base was the largest for external causes of death but, at the ICD-10 chapter level, the risk of death was higher for mental and behavioural disorders, injury, and poisonings. For some causes of death, associations were of a magnitude rarely seen in modern epidemiology [37]. We found no evidence of publication bias.

Our work is, of course, not without its limitations. We found evidence of heterogeneity between studies. The absence of agreed definitions of homelessness may explain some of this variation. Other sources of heterogeneity that we identified included stronger associations in studies using external comparators, of low quality, and lacking adjustment. To reduce the risk of confounding, we selected the most comprehensively adjusted estimate and, if multiple comparator populations were used, we selected the socio-economically disadvantaged. The main pooled estimate may therefore be an underestimate. We conducted a meta-analysis despite heterogeneity, as the effect of exposure to homelessness across all of its manifestations is arguably the most policy-relevant estimate. No studies were retrieved from low-income countries, including countries in Africa. The effects of homelessness on mortality in these contexts are therefore largely unknown. Lastly, exposure to homeless, a time-varying exposure, was measured only once in the studies. Because people are likely to transition in and out of stable housing [38], our results might be subject to some misclassification and have underestimated the true magnitude of the association.

Many of the individual causes of death that we identified in people exposed to homelessness—psychoactive-substance-use disorder, drug overdose, and alcohol-related—are avoidable [39]. Similarly, drug misuse and alcohol misuse are likely to be implicated in other causes of mortality that we found, such as transport accidents, external causes of accidental injuries (e.g. drowning, falls), and other accidents (e.g. accidental poisonings). This would suggest that access to evidence-based treatments for substance use, such as methadone [40], standard or long-acting buprenorphine maintenance therapy [41, 42], the distribution of naloxone to treat opiate overdose [43], and psychosocial interventions [44], may help ameliorate some of these excess deaths. Indeed, integration of these measures into rapid-response housing schemes such as Housing First, where housing is provided before issues such as mental illness or substance use are treated, have been associated with increases in the number of days in stable housing and hospital attendances [45]. Housing First has not, however, been associated with improvements in mental health or quality of life, and reductions in substance use [46]. In parallel, the lack of social housing and affordable private accommodation [47, 48] is a structural barrier that urgently needs to be addressed to enable policy responses to house people and support them to remain housed.

In conclusion, our meta-analysis shows that homelessness is associated with around a doubling of the risk of all-cause mortality. The most extreme inequalities were found for deaths due to psychoactive-substance-use disorder, drug overdose, accidental injury, and alcohol-related causes, which may have an interrelated aetiology. This suggests that more investment and evaluation of the inclusion of health-policy responses integrating health, housing, and social services targeting these deaths and their causes are needed.

## Ethics approval

The Survey of Health, Ageing and Retirement in Europe (SHARE) cohort was approved by the Ethics Council of the Max Planck Society. The Health and Retirement Study (HRS) cohort was approved by the University of Michigan’s Institutional Review Board. Written informed consent was given by study participants before the start of data collection.

## Supplementary Material

dyag129_Supplementary_Data

## Data Availability

Individual-participant data from the SHARE and HRS cohort studies are available to download from the Inter-University Consortium for Political and Social Research (http://www.icpsr.umich.edu/icpsrweb/ICPSR/). The data and code for the meta-analyses can be found at GitHub (https://github.com/jameswhite1979/Homelessness-systematic-review).
